# Pathology and Practice *(Medical)*

**Published:** 1818-04

**Authors:** 


### PART III.

### MONTHLY SKETCH OF FOREIGN MEDICAL SCIENCE.

——— Sparsa Colligens.

### I. PATHOLOGY AND PRACTICE *(Medical)*.

1. *Lesions of the Organs of Circulation.*—Respecting the precise mode of formation of *Aneurism*, considerable diversity of opinion has long existed among pathologists. Until the appearance of the magnificent work of Scarpa * upon this subject, the distinction of

* *Sull' Aneurisma, Reflessioni ed Osservazioni Anatomico-Chirurgiche.* See also the excellent translation of this splendid production, by Mr. Wishart. EDIT.

true and spurious aneurism, the one formed by simple dilatation of the arterial tube, the other, by rupture of the coats of the morbid vessel, and consequent effusion into the surrounding cellular structure, seems to have been generally agreed upon, and admitted, in the schools. Such opinions, however, the celebrated Professor of Pavia stepped boldly forward to denounce as erroneous. Aneurism, it is the object of his labours to represent as invariably formed by rupture or erosion of the unyielding internal and middle coats of an artery, and the dilatation of the external cellular coat by the blood consequently poured out into it. In confirmation of this doctrine, the testimony of preceding pathologists is very industriously collected and adduced; and, whenever such testimony does not exactly support his favourite notions, the Professor, with more ingenuity than fairness, ascribes the discrepancy to a defect of anatomical skill or correctness, on the part of those from whose descriptions he has been quoting. This, unfortunately for science, is no novel mode of procedure with theorists; but it is not more easy of accomplishment than ephemeral: and, in fact, it now seems acknowledged by our best and most experienced pathologists, that aneurism, formed by simple dilatation of the parietes of a vessel, although a form of lesion, comparatively rare with respect to that which results from a solution of continuity of the middle and internal arterial coats, is occasionally, and indeed not unfrequently, to be met with.* To the evidence already accumulated upon this point, the ensuing history † will form no uninteresting contribution.

A man, aged 45, and unmarried, of bilio-sanguineous temperament, robust constitution, and short stature, with black hair, and ardent passions, and much addicted to women, had endured many hardships while serving in the French army, and suffered much from domestic troubles. In June, 1816, after nine months' indisposition, he was obliged to exchange the active occupations, in which he had engaged at Paris, for a more sedentary life. In addition to the usual diseases of infancy, he had been successively affected with itch, gonorrhœa, and syphilis, and submitted to a methodical treatment for them. Previously healthy, he was first attacked in Sept. 1815, while walking, with tightness of breathing and palpitations in the centre of the thorax. Towards night, the pulsation was aggravated. Three days afterwards, pain in the chest was added to the palpitation and dyspnœa. Increased pain and difficulty of inspiration were thought to indicate bleeding; but this was rejected by the patient, who continued his occupations. In about two months, the pains became so severe as to disturb the

---

* See, upon this subject, the work of Mr. Burns on *Diseases of the Heart;* and that of Mr. Hodson on *Diseases of the Arteries.* The evidence of the former gentleman is very decisive. Ed.

† *Observation d'une Anévrisme de l'Aorte avant sa courbure; par M. H. F. Jannin, D. M.* &c. *Bulletin de la Société Médicale d'Emulation,* No. vii.—Juillet 1817.

sleep, and prevent much exercise. The case was now pronounced to be aneurism of the heart, and venæsection and abstinence were prescribed. General convulsions of the limbs, of some hours' duration, and an increase of the symptoms, followed the operation of blood-letting. It was, however, repeated, after some days, with a similar result; and, from derangement of the bandage of the arm, during the violent spasms which ensued, much blood was lost. Hence great debility was induced, but without relief of the dyspnœa, palpitation, or pain. The dietetic restrictions, meanwhile, were disregarded. Sedatives, and the application of leeches to the anus, were tried without effect. Dupuytren, consulted after some months, declared the disease to be aneurism of one of the principal arteries of the chest, and prescribed blood-letting, pediluvia, severe dietetic regimen, and tincture of digitalis internally; but the patient, ignorant of the character of aneurism, and destitute of confidence in his physicians, abandoned himself wholly to nature. Under these circumstances, in June 1816, Dr. Jannin was first summoned to the patient. His face was then somewhat swollen; his eyes prominent and watery; his lips large and bluish. He had a frequent sense of suffocation: his body was slightly but inalterably curved. The pulsations synchronous with, but perfectly distinct from, those of the heart, which occupied its wonted site, were perceptible above the right nipple, between the second and third sternal (true) ribs. The pulse of both sides, about 76 in a minute, evinced not the slightest irregularity. The patient, moreover, complained of acute thoracic pains, augmented by pressure. He walked but very slowly, and could not ascend stairs without resting himself, and being menaced with suffocation. Reclination on the sides was not long tolerable. He sat up through greatest part of each night, and slept very little. He was teazed with a dry convulsive cough. His appetite and digestive powers were unimpaired. An attempt was now made to dispel from the patient's mind the idea of aneurism; and a mixture of opium, digitalis, and sulphuric ether, prescribed with evident relief. Profound sleep and reduction of the pulse to 64 ensued: but, on discontinuance of the medicine from disgust, all the symptoms recurred with increased violence. The projected reapplication of leeches to the anus was frustrated by the inability of the patient to preserve long the same position; nor was it deemed necessary to urge the repetition of general blood-letting. From the prescription of conium with digitalis, and stimulant pediluvia, and the application of ice to the seat of the pulsation, some relief was now derived. Yet the progress of the disease was uncontrolled; and a tumour, with very strong pulsations, arose between the second and third sternal ribs. Reclination on the right side provoked a harassing cough. A transient respite from suffering was again afforded by the anodyne mixture; but, in two days afterwards, the pains became intolerable, and the pulse irregular, and, at times, imperceptible. On the 26th of October, Dr. Jannin found his patient with despair depicted on his countenance, and meditating self destruction. In order to inspire hope, fresh medicines were prescribed; and a poultice of digitalis leaves, sprinkled with camphor, applied to the aneurismal tumour. A pill of conium was also administered, and thus tranquillity in some degree restored. On the morrow, the aneurismal tumour burst during sleep. The patient had possessed sufficient strength to rise from his bed, reached a chamber-pot, into which he expectorated a large quantity of red frothy blood, and walked to the other extremity of his room, where he expired in a chair, with his head inclined backwards.

*Dissection.* The lungs were sound, but adherent to the ribs. The pericardium contained a considerable quantity of serum. The heart, although large, displayed no organic change. The aneurismal tumour, formed by dilatation of the aorta, anteriorly to its arch, adhered to the posterior part of the sternum, and the second, third, and fourth ribs. The anatomical preparation, after having been immersed in a saturated solution of muriate of quicksilver, exhibited the following dispositions:—The third sternal rib, partly corroded by the pulsations of the tumour, was perforated near its junction with the cartilage, which unites it to the sternum. In the compact substance of part of the inferior border of the second rib, and of the superior border of the fourth, the process of destruction had commenced. The aneurismal tumour, of an ovoid figure, presented in its long diameter, which had a direction from behind anteriorly, and somewhat from left to right, an extent of five inches from the paries of the aorta, opposite its dilatation, to the space comprised between the third and fourth sternal ribs. Its transverse diameter was four inches. The aneurismal tumour adherent, in a considerable extent, to the right lung, had burst near the entrance of the bronchiæ on the same side. The orifice, directed from behind anteriorly, and somewhat from above downward, was unequal, in the form of the italic *S*, and had an extent of two inches. The interior of the sac was strengthened, in its anterior portion, by thick albuminous layers, consisting of fibrine.

It is worthy of remark (we here quote nearly the words of Dr. Jannin, in confirmation of the strictures by which the present article is opened), that the aneurismal tumour, in this case, consisted not simply in dilatation of the external membrane of the aorta: the three coats evidently concurred in its formation; and, *by the aid of dissection, they were readily separated through the whole of its extent.* Nor did the tumour exhibit, at its base, that strangulation which is constantly observed in aneurism, with rupture of the proper coats of the vessel, nor any fissure of the artery in the interior of the sac; the cavity of which was evidently the same with that of the tumour. This could not have been the case, had the latter been formed exclusively by the cellular coat of the artery. Evidence like the preceding, no liberal and enlightened mind can reject. How decidedly it operates in refutation of Professor Scarpa's doctrine, we need not pause to explain.

Previously to our quitting the present section, we shall introduce another case of aortic aneurism,* complicated with dropsy, and a singular tendency to the deposition of calcareous matter. It has recently been observed by a medical student at the Parisian hospital *la Charité*. An old soldier, aged 66, of strong constitution and sanguineo-lymphatic temperament, formerly a sufferer from syphilis and rheumatism, which had never been properly treated, contracted a cold in December 1816, and utterly neglected himself. But, in January 1817, on the accession of hoarseness and slight dyspnœa, he entered *la Charité*. On the 12th, the following symptoms were noted: Face swollen, especially about the eye-lids and lower part of the head; the superior limbs and thoracic and abdominal parietes natural; inferior limbs œdematous; pulse not very frequent, unequal, and intermittent; tongue whitish and moist; appetite good; no unusual thirst; alvine discharges scanty; urine not sufficiently abundant; respiration slightly embarrassed; cough frequent, with difficult expectoration of mucus, swimming in much frothy liquid. On striking the chest, the region of the heart, and the lower part of the cavity, on each side, rendered an obscure sound, indicating effusion into the pericardium and sacs of the pleura. Examination of the abdomen disclosed neither the presence of fluid nor any trace of organic lesion. But, beneath the integuments, upon the internal aspect of each thigh, was felt a hard substance, commencing about two inches from the pelvic attachment of one of the inner femoral muscles, and extending to the middle of the limb. That of the right side was much the largest. These indurations, of the existence of which the patient had never before been aware, offered no obstruction to the movements of the thighs; nor did any pain result from forcible pressure upon them. Dr. Lerminier, of *la Charité*, considered the case to be one of lesion of the heart; and inferred, from the presence of these osseous concretions of the muscular system, that such lesion would prove to consist in ossification of the aortic valves, and of some portion of the aorta. Tonics and diuretics were prescribed. On the 14th, the œdema was augmented, and obscure fluctuation was perceptible in the abdomen. On the 16th, the presence of an abdominal fluid was more decidedly marked, and the chest sounded more obscurely than ever, on percussion. Diuretics were prescribed in an increased dose. Henceforth, the dropsical symptoms nearly disappeared; but they recurred on the 21st in an aggravated form, with complete loss of appetite, increased arterial irregularity and dyspnœa. The symptoms now grew constantly worse; the features changed; the strength sank; and the final struggle took place on the 27th.

*Dissection on the 28th. External appearances.* The face and

---

* *Ossification de quelques portions des muscles, et des valvules aortiques, avec anévrisme de l'aorte, hydro-péricarde, hydrothorax, ascite et anasarque, &c. &c.* Par M. M. Boncamp, &c. *Gazette de Santé*, Mars, 1817.

lower limbs much swollen; the trunk and superior limbs greatly emaciated. On examination of the left thigh, the indurated body, which had been felt during life, was found occupying the inferior portion of the middle adductor muscle, and imbedded in its substance. It was two inches in length, and one in diameter, and could not be separated from the muscle without division of the fibres. Its physical characters strongly resembled those of bone. It terminated abruptly, without the intervention of cartilage between it and the fleshy fibres above, and the tendinous below. The first adductor of the right limb presented an ossification much more considerable than the preceding. Commencing near the inferior attachment of the muscle, it extended to the middle; and, like the other, exhibited numerous small holes resembling the nutrient orifices of bone. In the interior was, moreover, presented a kind of net-work, perfectly similar to that of cellular bones. ***Head.*** Dura mater perfectly adherent to the cranial bones; brain firm; about one ounce of serum in each ventricle, and two ounces in the inferior occipital fossæ: cerebellum soft, with a greyish white surface. *Thorax.* Two ounces of bloody serum in the pericardium; heart voluminous, and covered, like the internal surface of the pericardium, with thick albuminous layers. The orifice of the aorta nearly surrounded with an osseous concretion, which appeared to belong rather to the muscular portion of the heart than to the commencement of the artery; the ossified aortic valves possessing little motion, and hence greatly contracting the aperture of the vessel: near the orifice of the subclavian arteries, a small aneurism, strongly adherent to the surrounding parts, which were themselves thickened and indurated. *The two internal arterial coats were deficient at the point occupied by the aneurism;** and the remnant of the vessel exhibited several specks of ossification. This was also the case with the crural, and several of the other arteries of the limbs. About one *pinte* of reddish fluid in each sac of the pleura; the left lung small, adherent above, and, like the right, gorged in its posterior, but crepitous in its inferior portion. The bronchial membrane exhibiting a faint red colour, and smeared with a frothy liquid. *Abdomen.* Peritonæum containing two *pintes* of a clear yellow fluid. The spleen of an ordinary volume, exhibiting in the centre of its external surface a superficial ossification, by which it was firmly connected to the diaphragm. All the other abdominal organs sound.

Pathology and Practice *(Surgical).* Much has recently been said upon the employment of *Gastrotomy* for the removal of

---

* Here the difference of morbid structure from that of the preceding case is very obvious. This, in fact, offers an example of aneurism, illustrating the theory of its formation too exclusively maintained by the Professor of Pavia.—Ed.

intus-susception, and other obstructions of the intestinal canal, arising from a mechanical cause, and unmanageable by the more lenient and ordinary means which experience can suggest, or the art of medicine supply. Some very daring and extraordinary efforts of this description have, within the last few months, come to our knowledge; but their issue, we lament to say, has generally, if not invariably, been such as to inspire no very sanguine expectations with respect to the ultimate fate of this bold chirurgical experiment.

By a French surgeon of the name of Monfalcon, the question of the applicability of gastrotomy to cases of otherwise irremediable obstruction of the intestinal tube has lately been discussed.* And we are so much pleased with the order, clearness, and ability, which characterize his production; and so warmly interested by the valuable pathological facts with which it abounds, that, assured of the general participation of our readers in these sentiments, we shall proceed to give a full and correct analysis of M. Monfalcon's Memoir.

To examine whether Gastrotomy ought to be practised in internal strangulations of the intestinal canal; to arrange such strangulations; to controvert the plea for a dangerous and unduly frequent operation; and, finally, to record a singular and important case connected with it, constitute the objects of this dissertation.

The numerous causes from which internal strangulations may arise, furnish a basis for the establishment of the following species:

1st. *Internal Strangulation from a twist of the Intestine.*—Intense ileus sometimes accompanies this unnatural state, and opposes an insuperable obstacle to the passage of the fæces. Simson has constantly seen, in subjects destroyed by volvulus, high inflammation of all the intestinal membranes. In one instance, he found the ileum, of a deep-red colour, thrust into the cæcum and colon, which were transposed from right to left; and all the parts were firmly agglutinated together. The ileum of another individual was, in three or four places, reflected on itself; and general and intense enteritis prevailed. Scarpa has frequently seen the intestine so twisted in the 8 form, that he could not distinguish the superior from the inferior end; and thinks this a more common occurrence than is generally supposed; whether it takes place as the intestine passes from the ring, or commences after the hernia has acquired a certain volume.

2d. *Internal Strangulation from adhesion of an intestinal Appendix.*—A rare example of this species of strangulation is consigned by Dr. Regnault, in the *Journal Universal des Sciences Médicales.* It was formed by an appendix of the small intestine,

---

* *Histoire d'une operation de gastrotomie, faite pour detruire un étranglement interne, &c.* Par J. B. Monfalcon, &c. *Recueil périodique de la Société de Médecine de Paris,* Aout, 1817.

about seven inches long, which had completely obliterated the intestinal canal by a knot including a portion of mesentery.*

3d. *Internal Strangulation from adhesion of the Appendix Vermiformis.*—Scarpa, on opening the body of a young man, who had died from ileus, found this appendix much elongated, adherent, by its summit, to the cæcum, and forming a ring which embraced, and strangulated, the small intestine. These strangulations, from adhesion of the appendix vermiformis, are not uncommon. Joyand discovered, on dissection of a soldier, who had fallen a victim to the iliac passion, a species of internal hernia, formed by a portion of the ileum, about twenty-two inches long; which had slipped under the appendix of the cæcum, through its membranous band, and was so straitly confined in the passage that the finger could not be introduced without forcing the orifice.

4th. *Intus-susception of the Intestine.*—Of this disease, otherwise called volvulus, from the Latin word *volvere*, the ileum constitutes the ordinary seat. It has received, from various writers, different denominations. A curious case of it has been published by Garengeot. Hevin has shewn the powerful efforts made by Nature in this terrible disease. We have known it strike with death, and cause to be expelled by the anus, twenty-three inches of the colon, with its attached portion of mesentery; twenty inches of small intestine; and the whole cæcum with six inches of colon and ileum;—as in the cases respectively recorded by Lobaux, Salguer, and Fauchon. The nature of the malady is well expressed by the term, Intus-susception.†

5th. *Internal Strangulation from Stricture of the Intestine without known Cause.*—This variety has been observed by Lafaye on the body of a soldier, who had been destroyed by violent colic. The inflamed intestines were excessively distended by air and fæcal matter. At the point of union of the colon with the rectum, about the angle formed by the last lumbar vertebra with the sacrum, the contraction of the intestinal canal was such as not to admit the extremity of the little finger. On examination of the interior, the intestine looked as though it had been strangulated by a thread-ligature. The stricture had allowed the introduction of the fluid of glysters, but obstructed their return. A similar case has been recorded by Charve.

6th. *Internal Strangulation from a scirrhous Tumour developed in the Substance of the intestinal Parietes.*—Castanet found in the arch of the colon of a woman, who died from violent colic, with which she had been for several months tormented, a large tumour including the coats of the intestine. The stricture of the canal had

---

* For a nearly similar case, see *Med. Chir. Journal* for Nov. last. Ed.

† See Article *Ileus*, *Dictionnaire des Sciences Médicales*.

retained, above the tumour, a mass of fæcal matter by which the colon was considerably distended.

7th. *Internal Strangulation from a Slip of Omentum.*—This may take place in different ways. Sometimes a portion of omentum contracts immediately behind the abdominal ring, and assumes the form of a small cord, which, adhering strongly to a point of the abdominal parietes, crosses the intestine, and attaches itself to some other point of the abdomen, more or less remote from the first insertion. This fibrous band of omentum fails not to become a cause of strangulation, even when it does not completely circle the intestine, as often as the latter is distended by the development of gas, or accumulation of fæcal matter. At other times, a portion of omentum, adhering to the neck, fundus, or sides of an hernial sac, unites towards the centre into a single longitudinal band, which passes over the middle of the intestine, and divides it into a right and left part. These omental bands are sometimes situated at a considerable depth in the abdomen. Their existence behind the ring, enforces the important precept of introduction of the finger into the abdomen in the operation for hernia.

8th. *Internal Strangulation from twisting of the Omentum round the Intestine.*—This species is much less rare in hernia than in the interior of the abdomen.]

9th. *Internal Strangulation from Adhesion of the Omentum or Mesentery.*—These organs sometimes contract adhesions with the peritonæum or intestines. If the loop of intestine, engaged beneath the adherent portion of omentum, be strongly compressed from swelling of the digestive tube during an attack of peritonitis, or from other distending cause, internal strangulation will supervene. Of this an example will be presently detailed.

10th. *Internal Strangulation from the Passage of a Loop of Intestine through a Rupture of the Omentum.* Instances of this fatal accident are not rare. It may result from a fall or violent effort; but is more particularly observed in hernia. It has been known to occur, during the pains of parturition, in a woman affected with intestinal hernia. In entero-epiplocele, the intestine is commonly situated behind the omentum; and sometimes this membrane, contracting adhesions with the sides and fundus of the hernial sac, forms a sort of bag in which the intestine is enclosed. If the intestine be thrust violently against the omentum, it may rupture the latter, pass through it, and thus become strangulated by a real membranous ring. Scarpa has seen the omentum traversed by a knuckle of intestine in the inguinal hernia of a man, by whom no symptoms of strangulation had been experienced; although the thickness and induration of the borders of the omentum bespoke a rupture of long standing.

11th. *Internal Strangulation from the Passage of Intestine, through the Diaphragm into the Thorax.* For an illustration of this species, we refer the reader to an interesting article on diaphragmatic rupture, in the French Dictionary of Medical Sciences; of which a full account has recently been given in this Journal.*

12th. *Internal Strangulation from the Passage of a Loop of the Intestine into the Portion of Peritoneum forming the Tunica Vaginalis, and the Displacement of the Testis.* An instance of this extraordinary species, described by Fages,† will be hereafter noticed.

13th. *Obliteration of the Intestine by a foreign Body.* This substance may consist of bone or indurated fæcal matter. A young man, aged about 20, with the design of repressing an obstinate diarrhœa, imprudently ate a large quantity of hard eggs. The constipation, which succeeded, was insuperable; and the incessant vomiting was only terminated by death, which happened in a few days. The intestines were prodigiously distended between the stomach and a column of indurated fæces in the jejunum. This is not properly a case of internal strangulation; but still, as requiring the same operation as the preceding, it is ranged among them.‡ Obliteration of the intestinal canal may also be caused by the development of a tumour in its vicinity. Other causes, belonging to the history of external strangulation, fall not properly within our notice here.

*Signs of Obliteration of the intestinal Canal from an internal Cause.* Painful tension of the abdomen; insuperable constipation; hiccup; nausea, and inclination to vomit; vomiting of matter, at first chyly, then undigested, and lastly stercoral; extreme general uneasiness; pulse small and wiry; face bathed in clammy perspirations, and, like the extremities, cold; decomposition of the features; hollowness of the eyes; great distension of the abdomen, and pain in some determinate point of the cavity, previously to, and during, these symptoms; feeble respiration; continual sleeplessness; sometimes momentary amendment; death.

The pain, invariably fixed in one point of the abdomen, indicates the seat of strangulation; but it is necessary that the pain should manifest itself suddenly, and precede peritonitis. If it move from place to place; if it be insensibly developed; and the physician be ignorant of its origin, he can acquire no certainty as to the nature of its cause. This pain is rarely acute, but accompanied by a distressing sense of constriction and of weight.

In general, the existence of internal strangulation is readily ascertained; but its peculiar species and precise situation can rarely be distinguished. Volvulus of the jejunum has, in one instance, remained undetected till after the death of the subject. Strangu-

---

* See *Medico-Chirurgical Journal and Review,* vol. iv. page

† *Recueil périodique de la Société de Médecine de Paris,* tome vii.

‡ For an interesting account of two cases of death, from inordinate ingestion of eggs, by Mr. Morrison, see *Med. Chirurg. Journal,* vol. iv. page 7.

lation may be inaccessible to the hand of the surgeon, while the pain exhibits all the characters just indicated: nor is there any thing in this symptom which will authorize a prudent surgeon to undertake the dangerous operation of gastrotomy.

The Memoir of M. Monfalcon will be concluded in our next Number.

